# Label-free isolation and deposition of single bacterial cells from heterogeneous samples for clonal culturing

**DOI:** 10.1038/srep32837

**Published:** 2016-09-06

**Authors:** J. Riba, T. Gleichmann, S. Zimmermann, R. Zengerle, P. Koltay

**Affiliations:** 1 Laboratory for MEMS Applications, Department of Microsystems Engineering-IMTEK, University of Freiburg, Georges-Koehler-Allee 103, 79110 Freiburg, Germany; 2 Hahn-Schickard, Georges-Koehler-Allee 103, 79110 Freiburg, Germany; 3 BIOSS – Centre for Biological Signalling Studies, University of Freiburg, 79110 Freiburg, Germany

## Abstract

The isolation and analysis of single prokaryotic cells down to 1 μm and less in size poses a special challenge and requires micro-engineered devices to handle volumes in the picoliter to nanoliter range. Here, an advanced Single-Cell Printer (SCP) was applied for automated and label-free isolation and deposition of bacterial cells encapsulated in 35 pl droplets by inkjet-like printing. To achieve this, dispenser chips to generate micro droplets have been fabricated with nozzles 20 μm in size. Further, the magnification of the optical system used for cell detection was increased. Redesign of the optical path allows for collision-free addressing of any flat substrate since no compartment protrudes below the nozzle of the dispenser chip anymore. The improved system allows for deterministic isolation of individual bacterial cells. A single-cell printing efficiency of 93% was obtained as shown by printing fluorescent labeled *E. coli*. A 96-well plate filled with growth medium is inoculated with single bacteria cells on average within about 8 min. Finally, individual bacterial cells from a heterogeneous sample of *E. coli* and *E. faecalis* were isolated for clonal culturing directly on agar plates in user-defined array geometry.

Increasing interest in single-cell analysis throughout life sciences and industry^1^,^2^,^3^,^4^ within recent years raised the demand for tools to sort, isolate and handle individual cells. Although the majority of published single-cell studies is based on analysis of mammalian cells, single-cell analysis of microorganisms and especially prokaryotic cells comes more and more into focus^5^,^6^.

The ability to extract genome sequences by DNA amplification from a single cell has already led to a new paradigm in the analysis of complex microbial samples in addition to the metagenomics approach. Now, unculturable microorganisms, which represent the vast majority of the microbial world and are estimated to comprise up to 10^12^ mostly still unknown species^7^, can be characterized from a single individual cell^8^. This allows not only for phylogenetic classification but has also led to the discovery of new genes and their functions within the so called “microbial dark matter”^9^. Especially the isolation of bacterial strains from inhospitable habitats like toxic waste, anaerobic environments or matter of high osmotic pressure or temperature often fails due to the need for precisely controllable culturing conditions. On the other hand, studies have shown that such an effort will be worthwhile by the identification of new classes of enzymes, which e.g. enable the degradation of environmental toxins^10^. Bypassing culturing by direct single bacteria sequencing from heterogeneous populations thus expands the toolbox to access such information and complements the metagenomics approach.

Industrial biotechnology production is carried out using wild-type or genetically modified pathways of certain yeast, fungi or bacteria to enrich the product of interest within a batch or fed-batch process. In the same way, many non-synthetic drugs are often produced using recombinant strains. In accordance with the major goal of batch-fermentation to maximize product yield, the search for best producers is an ongoing process. Irrespective of the genetic optimization of pathways (which is often accompanied by random mutagenesis) or the *de novo* screening for microorganism expressing e.g. new antibiotics, the isolation of pure strains for identification is usually required. To the best of our knowledge, this is still carried out manly by the classical method of spreading a cell suspension or environmental sample on nutrient agar plates followed by cultivation and clone picking. The demand of automation in high-throughput screening thus was addressed by the development of colony-picking robots able to map out colonies on agar in common petri dishes and isolate cells from those colonies into liquid broth. Therefore strain isolation still requires either laborious manual procedures or the use of expensive automation equipment.

Due to the intrinsic phenotypic heterogeneity even within clonal cell populations, it became clear that studies addressing fundamental cellular behavior and functional analysis on the single-cell level can provide so far inaccessible information. In this line, single-cell growth studies revealed an increased growth rate compared to common batch cultures^11^ and the relation of glycolytic oscillations in yeast cells and their synchronization was demonstrated^12^. These and similar studies call for further technologies to sort and isolate individual cells for single-cell analysis.

In this context, Rinke *et al.* established a workflow for sequencing of single microorganisms by sorting fluorescently labeled cells with a fluorescence activated cell sorter (FACS) into 384-well plates followed by whole genome amplification (WGA)^13^. The workflow was utilized for sequencing and genotyping of yet uncultured marine microorganisms^9^. Droplet microfluidic approaches for isolation of microorganisms and subsequent molecular analysis have been explored for similar purpose as well^14^. However, up to date the isolation of single microorganisms in droplet microfluidics is based on statistical encapsulation governed by the Poisson distribution, which lacks a direct proof of clonality and results in a large number of empty droplets. Though droplet microfluidic devices are usually operating at high throughput, individual single cell-containing droplets cannot be easily retrieved from the microfluidic chips limiting the usability of a specific microfluidic chip design to one single application. Optical tweezers were applied to overcome the statistical nature of the cell separation process by manually selecting single cells from sediment samples in a microfluidic chip mounted on an inverted microscope^15^. Albeit providing a higher control in the cell selection process, manual cell sorting limits the degree of automation and thereby the throughput.

In our previous work, a single-cell printer (SCP) was presented, which allows for sorting, isolating, and depositing of individual mammalian cells of 10–25 μm in size in a label-free and non-contact manner^16^. This approach complements the previously mentioned technologies such as FACS, microfluidic devices or manual single-cell manipulation to some extent as it operates label-free and provides a high degree of automation as well as flexibility regarding the application and analysis method. For the present work, the optical detection system of the previously presented SCP prototype instrument^16^ was redesigned. Further, dispenser chips with smaller channel depth and nozzle were fabricated to allow for detection of cells down to 1 μm in size. The modified SCP prototype can be considered as a generic platform to isolate and deposit individual bacterial cells onto any given substrate such as microwell plates, microscope slides or agar plates. In this article ink-jet like drop-on-demand printing of individually selected bacterial cells is presented for the first time. The performance of the modified SCP prototype is evaluated by deposition of fluorescently labeled *E. coli* cells and finally the instrument is used to separate individual cells from a heterogeneous sample for clonal culturing in micro-wells and onto agar plates in user-defined patterns.

## Results

### Single-cell detection optics and dispenser chips for bacterial cells

**Figure 1:**
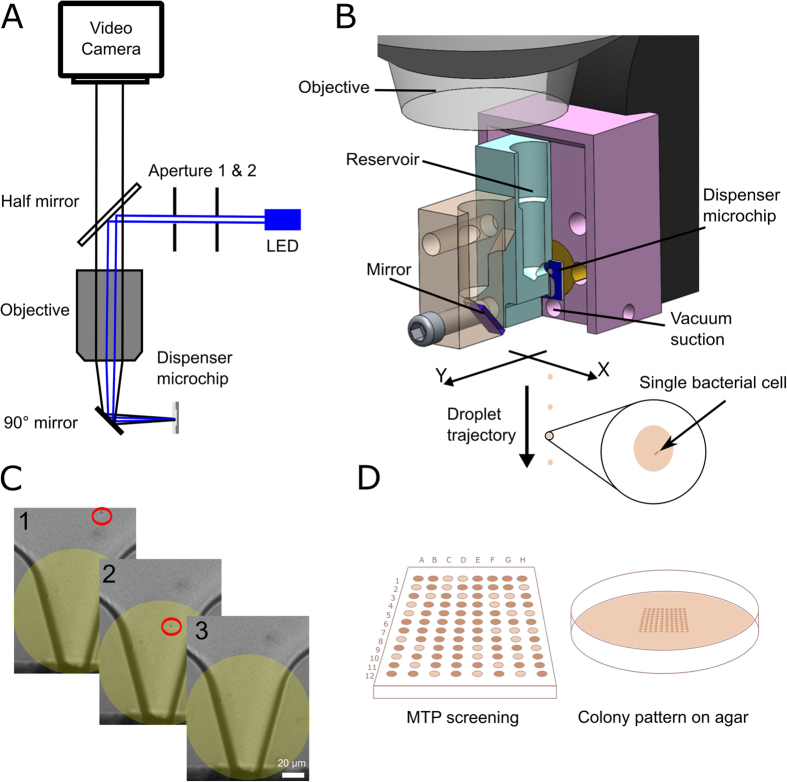
Principle of Single-Cell Printing. (**A**) shows a scheme of the bright-field optical detection system as implemented in this work. Light of a high-power blue LED passes two apertures and illuminates the dispenser chip through a 10x objective. Light rays reflected from the chip pass a half mirror and are detected by a high-resolution camera to image the dispenser chip nozzle. The single-cell printhead (**B**) comprises a piezo-stack actuator, the optical detection system, and the disposable cartridge including a sample reservoir and the microfluidic dispenser chip. A small mirror in front of the dispenser chip allows for tilting the optical path by 90°. On actuating the piezo-stack actuator, the piston displaces a constant volume within the chip generating a single droplet of 35 pl ejected from the nozzle. An algorithm coupled to the optical feedback of the camera decides whether the volume is expected to contain a single cell to print the droplet to the target or to remove it otherwise by a vacuum suction. A consecutive image series (**C**) from each single printed cell is stored on the PC. In this study the instrument was used for single-cell patterning on agar plates and deposition of single cells into a microwell plate like illustrated (**D**).

The working principle of the SCP as previously published in detail^16^ is shown in Fig. 1B. Briefly, droplets are generated on-demand from a micro-machined dispenser chip upon deflection of a silicon-membrane with a piezo-actuated piston. The nozzle region of the dispenser chip is continuously monitored by a video microscope which provides the image data for the cell detection algorithm that classifies the number, size, and morphology of the objects that are predicted to be ejected with the subsequent droplet. A vacuum-shutter system allows for the removal of unwanted droplets after ejection from the nozzle (i.e. void droplets or droplets with multiple cells) before they can reach the target substrate. For each deposited droplet a series of images of the nozzle region is automatically stored comprising an image before, at and after the cell is ejected, respectively. These images can be reviewed manually by the operator after the cell isolation process in order to verify whether truly a single cell was ejected with each droplet (Fig. 1C and Supplemental Fig. S1). Compared to most mammalian cells-usually larger than 10 μm in size-bacterial cells are found in dimensions down to the sub-micron scale^17^. As the cell detection of the SCP is based on bright-field imaging, the resolution of the detection optics had to be increased. This was achieved by using an infinity corrected microscope objective (M Plan Apo 10x, Mitutoyo, Japan) with NA = 0.28 and a 215 mm lens barrel resulting in a total magnification of 11.7x. A working distance of 33.5 mm enables to tilt the light path by 90° using a mirror integrated into the fixture for housing the dispenser chip to image the nozzle region from the top (see Fig. 1A–C). This ensures collision-free addressing of any flat substrate (Fig. 1A) because no compartment protrudes below the nozzle of the dispenser chip anymore. The sensor of the monochrome digital camera (UI-3480CP, IDS Imaging, Germany) has a pixel size of 2.2 μm resulting in a projection scale of 0.19 μm/px. This yields a theoretical spatial resolution of 0.38 μm/px. Hence, the performance of the system is not limited by the “digital resolution” of the sensor or the magnification, but rather by the optical resolution of the optical lenses which can be estimated by the Rayleigh criterion d = 0.61 λ/NA yielding d = 1.01 μm using a blue LED (λ = 465 nm) for illumination.

**Figure 2:**
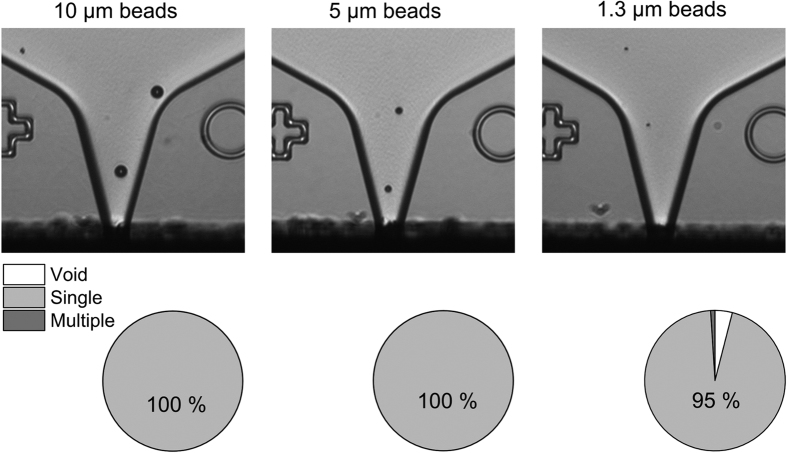
Detection and isolation of beads ranging from 10 μm to 1.3 μm in size. Fluorescently labeled beads were printed in 10 × 10 arrays on a glass slide using dispenser chips with 20 μm nozzle size. The upper panel shows images of the chip nozzle with 10 μm, 5 μm, and 1.3 μm latex beads, respectively. Single-bead printing efficiency was evaluated with a fluorescent microscope yielding 100% for the 10 μm and 5 μm beads, and 95% for the 1.3 μm beads (lower panel).

The contrast of the cell images could be significantly increased by collimating the incoming light from the blue LED with two apertures. Since the focal depth of the objective yields only 3.5 μm, the chamber and channel depth of the dispenser chips was decreased from 40 μm to 20 μm. The reduction of the nozzle size associated with the smaller channel depth results in reduction of the droplet volume from 150 pl to 35 pl which was quantified by stroboscopic imaging and a gravimetric method described by Liang *et al.*^18^. Combining smaller dispenser chips with an improved detection optic allows for the detection and isolation of objects ranging from 10 μm down to 1.3 μm which was demonstrated by printing 10 × 10 arrays of individual fluorescently labeled beads (N = 100 beads of each size). The single-bead printing efficiency was evaluated by microscopic analysis of the printed arrays and yielded 100% for both 10 μm and 5 μm sized beads, and 95% for beads 1.3 μm in size (Fig. 2).

### Single-cell printing efficiency can be evaluated by printing fluorescently labeled bacteria cells

**Figure 3:**
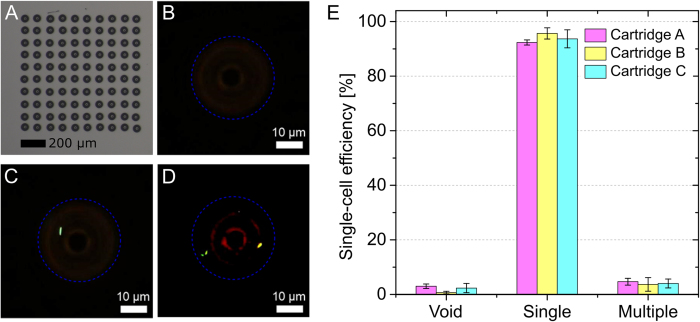
Single-cell printing efficiency was evaluated by printing GFP expressing *E. coli* cells on glass slides. Each spot of a 10 × 10 array (**A**) was classified via bright-field and fluorescent microscopy either as void droplet (**B**), droplet containing a single cell (**C**) or droplet containing multiple cells (**D**). The experiment was performed three times for each of three different dispenser chips. (**E**) The relative occurrence of events is plotted for each of the three tested dispensing chips. The corresponding CV was calculated based on the standard deviation of three repeated experiments. In total, 845 out of 900 spots produced in 3 × 3 experiments contained a single-cell, resulting in an averaged single-cell printing efficiency of 93%.

Single-cell printing efficiency, chip-to-chip performance as well as run-to-run performance as defined previously^16^,^19^ were investigated by printing three arrays (M = 3) each with three different dispenser chips (L = 3). One array consists of N = 100 spots deposited with a pitch of 100 μm on a glass slide. To evaluate the content of the droplets a suspension of fluorescently labeled *E. coli* cells in LB medium with 5% (v/v) glycerol was used for printing. After deposition of cell-encapsulating droplets the water evaporates within seconds while the remaining glycerol spots (Fig. 3A) allow for localizing and counting the number of cells in each droplet using phase contrast microscopy. Fluorescence microscopy was used to verify whether the printed object is indeed an *E. coli* cell (Fig. 3B–D). The single-cell printing efficiency-defined as the ratio of spots containing a single cell and the total number of spots printed onto the substrates-for the three dispenser chips averaged over M runs yielded 92 ± 1%, 96 ± 2%, and 94 ± 3%, respectively. The results are summarized in Fig. 3E. Averaging over all L chips and M runs results in an average single-cell printing efficiency of 93 ± 1.4%. Out of all printed spots 4 ± 0.4% contained multiple cells and in 2 ± 1% no cell could be identified by microscopy. The question arises whether these incorrect events are reflected by their SCP image series. Therefore, all SCP images where reviewed manually for comparison with the data obtained from the microscope. Indeed, the majority of the incorrect events could have been predicted by manual assessment of the SCP images. In 1.4% of all printed spots fewer cells were identified by microscopy than what can been seen on the respective SCP images. That means, although one (or two) cell-sized objects are visible on the nozzle images stored by the SCP, no (or one) cell could be identified in the printed spot under the microscope. On the other hand, 0.4% of all printed spots contained more cells than SCP images have shown.

### Single bacterial cells isolated with the SCP can be grown to clonal culture

**Figure 4:**
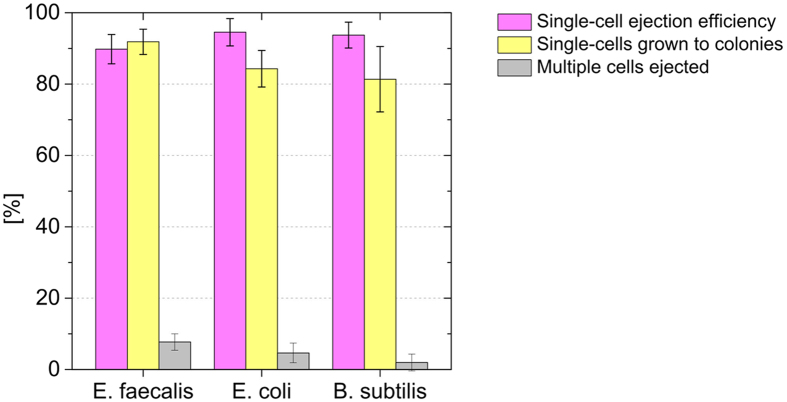
Clonal cultivation in microwells. Individual cells from three different species were deposited into microwells of 96-well plates (N = 10 for each species) filled with 200 μl culture medium. The SCP images were reviewed to determine the fraction of dispensed single-cells (ejection efficiency, magenta). After incubation for 36 hours, wells containing bacterial cultures were counted. To determine the number of single cells grown to colonies (yellow), only wells were a single cell was deposited were taken into account. The fraction of ejected droplets that contained multiple cells is also depicted (grey). The error bars refer to the standard deviation between individual 96-well plates.

Further experiments have been performed in order to assess the potential of individual printed bacterial cells from various species to grow to culture in liquid nutrient medium. Therefore, cells from homogeneous cultures of *E. coli*, *E. faecalis* and *B. subtilis* were deposited into microwells filled with 150 μl lysogenic broth (LB) medium. For each species, ten 96-well plates were inoculated (N = 960 wells) taking on average 8.1 ± 1.8 minutes to process a single 96-well plate. After incubation for 36 hours the number of wells in which a bacterial culture had grown was counted to determine the fraction of occupied wells on each plate (see Supplemental Table S1). The SCP images were examined to determine the single-cell ejection efficiency-defined as the ratio of ejected droplets containing a single cell and the total number ejected droplets targeted to the substrate-yielding on average 93% and to assess whether truly a single bacterial cell was deposited into a given well. From these data the fraction of printed single cells that were grown to culture was calculated, yielding 92% (*E. faecalis*), 84% (*E. coli*), and 75% (*B. subtilis*). The fraction of ejected droplets containing multiple cells was 8% (*E. faecalis*), 5% (*E. coli*), and 2% (*B. subtilis*). The results are summarized in Fig. 4.

### Single bacterial cells can be isolated from a heterogeneous sample resulting in distinctive clonal cultures

**Figure 5:**
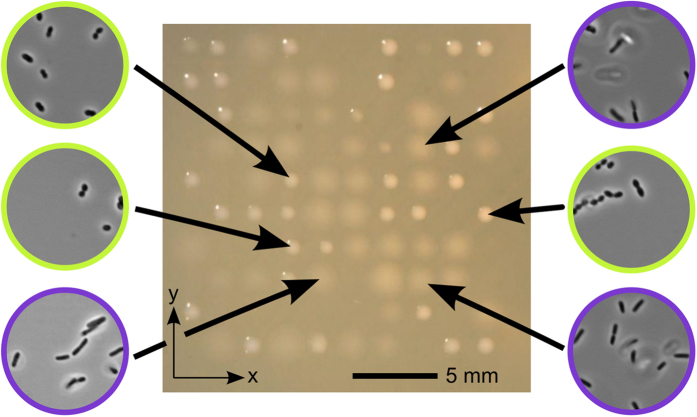
Bacteria colony array grown from 10 × 10 spotted single cells on LB-agar. Single bacteria cells were printed from a heterogeneous culture of *E. coli* and *E. faecalis* previously mixed in a ratio of 1:1. Obviously, two clearly distinguishable colony morphologies can be found for the two different types of bacteria. Visual inspection by light microscopy revealed that shiny sharped edge colonies were grown from *E. faecalis* (yellow circles) while mat colonies with diffuse edges could be assigned to *E. coli* (blue circles). In total, 71 out of 100 cells grow up to a colony with a ratio of 38:33 of *E. coli* to *E. faecalis*.

In order to demonstrate the instruments ability to isolate individual bacterial cells from a heterogeneous sample, we printed a binary mixture of cells of *E. coli* and *E. faecalis* at a ratio of 1:1. Both species grow under the same culture conditions and can be visually distinguished by light microscopy as they show a distinct morphology namely rod-like (*E. coli*) and cocci (*E. faecalis*). A 10 × 10 array of droplets with 2 mm pitch was printed directly on an LB-agar plate. After incubation for 12 hours at 37 °C, 71 out of 100 single cells grew to clearly visible colonies that could be assigned to two distinct morphologies and shapes as shown in Fig. 5. We randomly selected three colonies of each type of morphology and verified that they were either grown from *E. coli* (38 colonies) or *E. faecalis* (33 colonies) by light microscopic imaging.

## Discussion

Optical image detection of individual cells is an important premise to print single cells with the SCP-technology. By improving the bright-field optical system and using dispenser chips with a reduced channel depth and nozzle size it could be shown that the SCP is capable of detecting and isolating bacterial cells down to 1 μm in size. Since detection is not based on a fluorescent marker, bacterial cells can be isolated from homogeneous cultures (single species) as well as microbial communities of various species without any additional labeling steps. Such label-free detection furthermore avoids bias introduced by the labels itself 20.

Re-routing the optical path between the dispenser chip and the objective with a small mirror (Fig. 1) did not significantly impair the optical quality of the microscopic images and allowed for designing a printhead which can address the entire working space of the 3-axis robot without limits. With this approach, bacterial cells can not only be deposited into standard microwells but also in user-defined patterns on any flat substrate at coordinates that can be easily specified by the user via the SCP software interface.

The re-routed optical path enables to use of a microscope objective with a numerical aperture of 0.28. Although the optical resolution could be further increased employing a lens with even higher NA, we consider the current setup as the optimum since the tradeoff would be a smaller depth of focus and a shorter working distance.

A single-cell printing efficiency of 93% was achieved with the presented SCP prototype instrument and GFP-expressing *E. coli* cells. This is superior compared to any statistical isolation methods which result on average in 37% single-cells based on the Poisson distribution^21^. With the SCP configured for eukaryotic cell printing^16^ comparable single-cell printing efficiencies of up to 95% are achieved meanwhile routinely depending on the cell type and the culture conditions (unpublished data). Here, in rare cases (4%) multiple bacteria cells were printed onto the glass slides. Further, we noticed that more often multiple *E. faecalis* cells (7%) were ejected with the droplets compared to *E. coli* (5%) and *B. subtilis* (2%) as recognized from the SCP images (Fig. 4). This correlates with the observation that *E. faecalis* cells are smaller than *E. coli*, while *B. subtilis* is on average larger in size.

A reason causing ejection of multiple cells is that some cells are not properly detected by the automatic image recognition algorithm, which can be traced back to two phenomena. First, bacteria sometimes exhibit an optical contrast to low to be classified as a cell by the image processing algorithm if they are located not sufficiently close to the focal plane. And secondly, in some cases the cells cannot be detected because they are next to the walls of the microfluidic channel, a region, which is not sufficiently illuminated causing a shading of the object. Both issues are less severe for larger objects such as typical eukaryotic cells and could be addressed in the future by designing dispenser chips with an even smaller chamber depth to improve sharpness of the bacterial cells or by integrating a cell focusing mechanism^22^.

Almost all individual printed cells grew to culture in the presented experiments, although a slight variation among the three species was observed. Since the ejection efficiency is not significantly different amongst the species (Fig. 4) the observed variation is likely due to a biological reason rather than to the technical approach. An impact on cell viability by the printing process however cannot be fully ruled out based on the presented data. It would be therefore favorable to isolate bacterial cells from the given strains with an alternative method. Our effort to isolate and culture individual bacteria by hand-pipetting using the dilution-to-extinction method failed, since this is a statistical approach which requires the knowledge of the exact cell concentration prior dilution. Due to the small size and the fast generation time counting of bacteria cells (in cell counting chambers) was too error-prone for any quantitative measurement.

The ability to culture individual printed bacteria in microwells demonstrates that the SCP can be used to deposit single cells directly into growth medium for clonal culturing with a clonal yield per plate that is more than two-fold higher compared to what can be achieved by statistical cell isolation. Moreover, only in rare cases a well contains multiple cells and a manual assessment of the SCP images allow to exclude these from any further downstream analysis.

Finally, the presented experiments show that one can isolate individual bacterial cells from a heterogeneous sample followed by clonal culturing on agar plates using the SCP. This was demonstrated by printing cells from a binary mixture of *E. coli* and *E. faecalis* cells directly on agar.

In conclusion, we have designed and realized a single-cell printing prototype instrument specifically for the use with very small objects such as bacterial cells. Using this instrument it could be demonstrated, that the SCP technology enables the detection, isolation, and deposition of individual bacterial cells. Although the characterization was limited to cultured bacteria we believe that the instrument can be applied for environmental and clinical samples as well implementing a minimal sample preparation in advance similar as prior FACS sorting^13^. Large contaminating particles and bacterial clusters could be removed with a filter. Besides some exceptions^23^, bacteria are typically on the order of 1 μm to several microns in size^24^,^25^. This range can be well addressed with the new dispenser chips that have been fabricated for this work. Dispenser chips with a smaller nozzle size might be prone to clogging but could be useful for samples were only small bacteria (submicron to few micrometer in size) are expected and should be explored in the future.

The major advantages of the SCP technology compared to other methods is the sterile and disposable cartridge avoiding cross contamination, the flexibility of the instrument in terms of substrates and geometries, the ability to isolate bacterial cells without a fluorescent label, and the direct proof of clonality provided by the SCP images. The precise 3-axis system enables to place droplet encapsulated cells in user-defined geometries on planar substrates.

Although FACS sorters have been recently utilized for label-free sorting of bacterial cells based on forward and side scatter^26^, this approach requires extensive fine-tuning of the sorting parameters and might be limited to samples comprising species homogeneous in size. Droplet microfluidic approaches are in principle more suited for high-throughput applications requiring thousands of cells^27^,^28^ and droplets containing bacterial cells can be sorted based on extracellular metabolites^28^,^29^. However, a strategy for deterministic encapsulation of individual bacterial cells has not been demonstrated so far, likely since a direct proof of clonality would require either single-cell detection at very high frequencies during droplet generation or microscopic screening of temporarily stored droplets after cell encapsulation which would limit the throughput and might be to slow due to fast generation times of some bacterial species. Hydrodynamic cell trapping in microfluidic chips is used successfully for the isolation of tens to hundreds of mammalian cells^30^, however, a trap design is usually limited to a narrow range of cell diameters. With yeast cells 5 μm in size a single-cell trapping efficiency of 70% was realized^31^ and the fabrication of sub-micron features allows trapping of single *E.coli* cells^32^,^33^. However, addressing these different cell types required different trap geometries which are highly size selective and are therefore most likely not suitable for unbiased isolation of single bacterial cells from heterogeneous mixtures. Due to the small footprint, even complex single-cell analyses can be integrated on microfluidic chips^28^,^30^,^34^, but these are usually designed for a single assay. In contrast to these existing technologies, the presented SCP provides a very flexible tool for deterministic and proven cell isolation, is compatible with standard laboratory equipment, and is not limited to predefined downstream applications. Therefore, we believe that the SCP does not only complement the existing technologies but might be superior in cases where hundreds of single cells need to be isolated from heterogeneous microbial samples with high precision for further downstream analysis or clonal cultivation.

Future applications of the instrument include but are not limited to automated single-cell genomics of microbial cells from heterogeneous clinical or environmental microbial samples as well as single-cell growth studies. Furthermore, due to the modularity of the printhead we believe that this single-cell isolation technology can be integrated into other analytical instruments such as mass spectrometers for single-cell analysis.

## Materials & Methods

### Dispenser chip fabrication

Dispenser chips were fabricated in a clean room from 4” silicon wafers with 300 μm thickness. The fluidic chamber was 20 μm deep etched into silicon by deep-reactive ion etching (DRIE) after lithographic structuring of a positive photoresist. Through-holes in the glass wafer (pyrex) were fabricated by wet etching with 50% hydrofluoric acid (HF) using a 400 nm thick polysilicon layer as mask that was deposited by low pressure chemical vapour deposition (LPCVD). After anodic bonding of both wafers individual chips were obtained by dicing. Printing cartridges were finally assembled by adhesive bonding of the dispenser chips into a CNC-milled chip holder made from PMMA that is required for handling and mechanical fixing of the dispenser chips and also serves as reservoir for the cell suspension. Both, chip and holder undergo a thorough cleaning procedure including an oxygen plasma treatment to minimize organic contamination.

### Bacterial strains and culture conditions

Lysogeny broth (LB) was sterilized (autoclaved) and filtered by a 0.2 micron membrane to reduce impurities in size of the objects of interest. Shake flasks containing 50 ml LB were inoculated from glycerol stocks frozen at −80 °C (15% v/v glycerol) using sterile tips and incubated at 37 °C on a horizontal shaker (300 rpm). Wild-type *Bacillus subtilis* (strain DSM 4451) and *Enterococcus faecalis* (strain ATCC 29212) were both cultured in pure LB and collected while in their exponentially growth phase (OD_600_ ~ 0.4) to ensure maximal viability. The pBAD-GFP transformed *E. coli* strain BL21 was cultured in the same LB medium containing 100 μg/ml ampicillin (Neolab, Heidelberg, Germany). Green fluorescent protein (GFP) expression was induced after three hours of incubation adding 3% (w/w) L(+)-arabinose (Carl Roth, Karlsruhe, Germany) followed by incubation for another 3 hours prior to printing.

### Printing workflow

Prior printing cell concentration was determined via optical density measurements at 600 nm followed dilution to a final concentration of approximately 5·10^6^ cells/ml. Dilution was performed with sterile filtered LB medium if not otherwise stated. Typically 5–30 μl of the diluted suspension was pipetted into a sterile packaged cartridge. Dispenser performance was evaluated using a customized bottom-view camera setup: Single droplets are printed on a microscopic glass slide and observed by a camera from below for software-based droplet detection and stability characterization. Printing parameters were adjusted until droplets are constant in size and devoid of satellites. Typically the 20 μm dispenser chips were actuated at a downstroke velocity of 60–70 μm/s and a stroke length of 2.5–3 μm. Altogether, instrument preparation typically takes 5 minutes.

### Evaluation of single-cell printing efficiency

In order to evaluate the instruments performance, fluorescent microbeads of various size (Kisker Biotech, Germany) and GFP expressing *E. coli* BL21 were used for printing. The bacterial culture grown to OD_600_ ~0.8 was diluted in sterile filtered LB containing 5% v/v glycerol (Sigma Aldrich, Hamburg, Germany). Arrays of 10 × 10 droplets were printed with a pitch of 100 microns onto glass slides. By counting beads and *E. coli* cells via bright-field imaging and fluorescent microscopy (Olympus CKX 41) each spot could be classified into void droplets, single-cell containing droplets, or droplets containing multiple cells.

### Printing single bacterial cells for clonal culturing

Single cells from homogeneous cell suspensions were printed into 96-well microtiter plates containing 200 μl LB medium per well. The plates were sealed with Parafilm (Sigma Aldrich, Germany) to limit the evaporation of the culture medium prior incubation at 37 °C. After 12 hours, the Parafilm was removed followed by another 24 hours of incubation. The growth in microtiter plates was validated by OD_600_ measurements using a Wallac 1420 Victor^2^ plate reader. Binary heterogeneous samples were prepared by mixing *E. coli* and *E. faecalis* cultures of the same OD_600_ at printing dilutions followed by printing arrays onto LB agar plates and subsequent incubation for 24 hours at 37 °C. Finally, the agar plates were visually inspected for bacteria growth. Clonality of *E. coli* and *E. faecalis* was examined with oil immersion microscopy (Zeiss Axiophot).

## Additional Information

**How to cite this article**: Riba, J. *et al.* Label-free isolation and deposition of single bacterial cells from heterogeneous samples for clonal culturing. *Sci. Rep.* **6**, 32837; doi: 10.1038/srep32837 (2016).

## Supplementary Material

Supplementary Information
