# Supplementary material for: Label-free isolation and deposition of single bacterial cells from heterogeneous samples for clonal culturing
Source: Sci Rep. 2016 Sep 6;6:32837. doi: 10.1038/srep32837 (PMC5011771; doi:10.1038/srep32837)
Supplement: Supplementary Information [file srep32837-s1.pdf]

Supplemental material

Label-free isolation and deposition of single bacterial cells from heterogeneous samples for clonal culturing

J. Riba[†*,1], T. Gleichmann[†,1], S. Zimmermann[1], R. Zengerle[1,2,3], P. Koltay[1]

[1] Laboratory for MEMS Applications, Department of Microsystems Engineering - IMTEK, University of Freiburg, Georges-Koehler-Allee 103, 79110 Freiburg, Germany

[2] Hahn-Schickard, Georges-Koehler-Allee 103, 79110 Freiburg, Germany

[3] BIOSS – Centre for Biological Signalling Studies, University of Freiburg, 79110 Freiburg, Germany

* Corresponding author

[†] These authors contributed equally

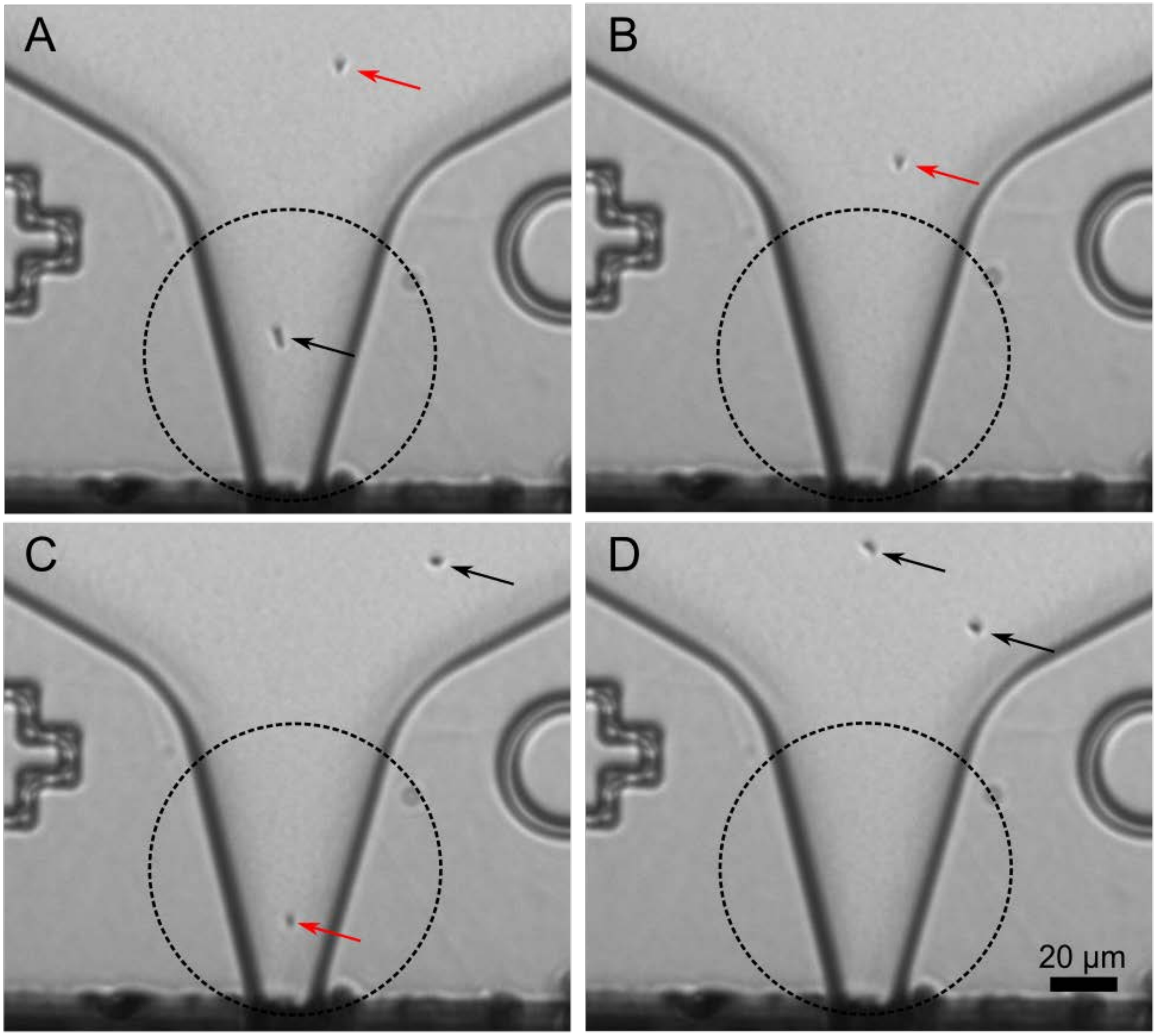

Supplemental Figure S1. The camera of the SCP is synchronized with the piezo actuator that generates the droplets. For each dispensed cell four images showing the nozzle of the dispenser chip are stored automatically by the instrument. The SCP images can be examined manually to assess whether truly a single cell was ejected from the nozzle. Here, a single *B. subtilis* cell (red arrow) is detected and printed. (A): image of the nozzle region two piezo actuations before detection, (B): one piezo actuation before detection, (C): at the time of detection, and (D): after the single cell has been ejected with the droplet. Only the droplet produced in (C) passes the vacuum shutter and is deposited on the substrate.

Supplemental Table S1. Cells of various species were deposited into 96-well plates for subsequent cultivation. The SCP images were assessed to determine whether a single cell, no cell, or multiple cells were ejected from the nozzle. The data is summarized in the manuscript in Figure 3.

Organism	MTP	No cell printed		Multiple cells printed		Wells occupied		Single cells grown to culture	
E.coli	MTP1	0	0.0%	1	1.0%	81	84%	80	84%
	MTP2	0	0.0%	1	1.0%	83	86%	82	86%
	MTP3	0	0.0%	1	1.0%	80	83%	79	83%
	MTP4	0	0.0%	7	7.3%	92	96%	85	96%
	MTP5	0	0.0%	3	3.1%	76	79%	73	78%
E.coli	MTP6	5	5.2%	5	5.2%	76	79%	72	84%
	MTP7	0	0.0%	6	6.3%	75	89%	69	88%
	MTP8	2	2.1%	9	9.4%	73	76%	64	75%
	MTP9	0	0.0%	6	6.3%	82	85%	76	84%
	MTP10	0	0.0%	5	5.2%	81	84%	76	84%
E.faecalis	MTP1	0	0.0%	5	5.2%	89	93%	84	92%
	MTP2	0	0.0%	12	12.5%	87	91%	78	93%
	MTP3	1	1.0%	8	8.3%	83	86%	75	86%
	MTP4	0	0.0%	7	7.3%	91	95%	84	94%
	MTP5	0	0.0%	6	6.3%	89	93%	83	92%
E.faecalis	MTP6	1	1.0%	4	4.2%	82	85%	77	85%
	MTP7	5	5.2%	7	7.3%	87	91%	80	95%
	MTP8	3	3.1%	9	9.4%	81	90%	73	94%
	MTP9	6	6.3%	6	6.3%	55	86%	50	96%
	MTP10	3	3.1%	5	5.2%	57	89%	51	91%
B. subtilis	MTP1	4	4.2%	1	1.0%	70	73%	68	75%
	MTP2	5	5.2%	4	4.2%	64	67%	59	68%
	MTP3	4	4.2%	4	4.2%	67	74%	64	78%
	MTP4	0	0.0%	0	0.0%	43	67%	43	67%
	MTP5	0	0.0%	7	7.3%	75	78%	69	78%
B. subtilis	MTP6	2	2.3%	0	0.0%	81	92%	80	93%
	MTP7	6	7.5%	0	0.0%	64	80%	64	86%
	MTP8	3	3.4%	1	1.1%	79	90%	78	93%
	MTP9	3	3.4%	1	1.1%	76	86%	75	89%
	MTP10	13	13.5%	0	0.0%	75	78%	72	87%
